# Prevalence of probable Attention-Deficit/Hyperactivity Disorder symptoms: result from a Spanish sample of children

**DOI:** 10.1186/s12887-018-1083-1

**Published:** 2018-03-15

**Authors:** Alberto José Cerrillo-Urbina, Antonio García-Hermoso, Vicente Martínez-Vizcaíno, María Jesús Pardo-Guijarro, Abel Ruiz-Hermosa, Mairena Sánchez-López

**Affiliations:** 1 Universidad de Castilla-La Mancha, Social and Health Care Research Center, Cuenca, Spain; 2 Laboratorio de Ciencias de la Actividad Física, el Deporte y la Salud, Facultad de Ciencias Médicas, Universidad de Santiago de Chile, Santiago de Chile, Chile; 3 Universidad Autónoma de Chile, Facultad de Ciencias de la Salud, Talca, Chile; 4 Universidad de Castilla-La Mancha, Faculty of Education, Cuenca, Spain; 5 Universidad de Castilla-La Mancha, Faculty of Education, Ciudad Real, Spain; 6 Universidad de Castilla-La Mancha, Edificio Melchor Cano, Centro de Estudios Socio-Sanitarios, Santa Teresa Jornet s/n, 16071 Cuenca, Spain

**Keywords:** ADHD, Attention Deficit Disorder with Hyperactivity, Attention Deficit Disorder with Hyperactivity/epidemiology, ADHD Rating Scale, Children, Socio-economic status

## Abstract

**Background:**

The aims of our study were to: (i) determine the prevalence of children aged 4 to 6 years with probable Attention-Deficit/Hyperactivity Disorder (ADHD) symptoms in the Spanish population; and (ii) analyse the association of probable ADHD symptoms with sex, age, type of school, origin (native or foreign) and socio-economic status in these children.

**Methods:**

This cross-sectional study included 1189 children (4 to 6 years-old) from 21 primary schools in 19 towns from the Ciudad Real and Cuenca provinces, Castilla-La Mancha region, Spain. The ADHD Rating Scales IV for parents and teachers was administered to determine the probability of ADHD. The 90th percentile cut-off was used to establish the prevalence of inattention, hyperactivity/impulsivity and combined subtype.

**Results:**

The prevalence of children with probable ADHD symptoms was 5.4% (2.6% inattention subtype symptoms, 1.5% hyperactivity/impulsivity subtype symptoms, and 1.3% combined subtype symptoms). Children aged 4 to 5 years showed a higher prevalence of probable ADHD in the inattention subtype symptoms and in total of all subtypes than children aged 6 years, and children with low socio-economic status reported a higher prevalence of probable ADHD symptoms (each subtype and total of all of them) than those with medium and high socio-economic status.

**Conclusions:**

Early diagnosis and an understanding of the predictors of being probable ADHD are needed to direct appropriate identification and intervention efforts. These screening efforts should be especially addressed to vulnerable groups, particularly low socio-economic status families and younger children.

## Background

Attention-Deficit/Hyperactivity Disorder (ADHD) is one of the most common neurodevelopmental disorders in children [1, 2]. According to the Diagnostic and Statistical Manual of Mental Disorders, fifth edition (DSM-5) [3], ADHD symptoms include difficulty staying focused and paying attention, difficulty controlling behaviour and hyperactivity. Three different presentations are recognised: the predominantly inattentive, the predominantly hyperactive/impulsive, and the combined presentation [3]. The etiology of ADHD is complex and multidimensional and combines environmental (e.g. home discord, low socioeconomic status, institutionalized care and exposure to violence and trauma) [4, 5] and genetic factors [6, 7].

Several studies confirm that ADHD symptoms cause a significant impairment in school tasks [8] and in the activities of daily life [9]. In most children with ADHD, symptoms persist into adolescence and adulthood, causing personal, social, occupational and even leisure time dysfunctions [10]; however, early diagnosis and appropriate treatment may positively influence this evolution [2] in a such way that many young people with ADHD are able to make a good adjustment to adult life and are free of mental health problems [11].

In epidemiological studies on the prevalence of ADHD it is necessary to distinguish various strategies: first, clinical (based on the assessment of an expert) and second, psychometric (based on scales of parents and/or teachers) [12]. There are several scales that meet the DSM-IV criteria for detecting ADHD symptoms, and in our opinion, one of the scales that best meets these criteria is the Attention Deficit Hyperactivity Disorder Rating Scales IV (ADHD RS-IV) [13] because of its reliability.

Estimates of the prevalence of ADHD in Spanish children and adolescents range between 4.9% and 8.8% [14]. Several factors have been described as responsible for this variability including the person reporting the ADHD symptoms (parent, teacher or child), the study methods and the diagnostic criteria used [14]. In addition, analysing the prevalence of each ADHD subtype is important and useful because each presentation is associated with different types of comorbid conditions [15]. Furthermore, it is also unclear whether the prevalence of ADHD and its subtypes is associated with certain population characteristics. Although it has been suggested that boys are more likely than girls to meet the criteria for an overall diagnosis of ADHD and for each of the DSM-IV subtypes [16], two Spanish studies showed that there were no statistically significant differences in ADHD prevalence between boys and girls [17, 18]. In addition to sex, a meta-analysis review shows that children of lower socio-economic status (SES) were 1.5–4 times more likely to meet the criteria for ADHD than individuals from families with high SES [16]. However, other studies have observed no difference among SES [19, 20]. Other socio-demographic factors, such as age [21, 22], nationality [23] and school type [24, 25] have been related to ADHD symptoms, but there is limited information on these factors in our context. Therefore, in our region, an understanding of the magnitude and predictors of being probable ADHD in preschool children is needed to direct appropriate identification and intervention efforts.

The aims of this study were two-fold: (i) to determine the prevalence of Spanish children aged 4 to 6 years with probable ADHD symptoms in the region of Castilla-La Mancha (Spain); and (ii) to analyse the association between that prevalence of children with probable ADHD symptoms with age, sex, type of school, origin (native or foreign) and SES.

## Methods

### Study population

This was a cross-sectional analysis of data (collected from September–November 2013) from a randomised cross-over cluster trial aimed to assess the effectiveness of a physical activity intervention (MOVI-KIDS) in preventing obesity and improving academic achievement in preschoolers with or without ADHD [26]. The MOVI-KIDS study included 1604 schoolchildren (aged 4 to 6 years) from 21 primary schools (19 public, 2 private) in 19 towns of Cuenca and Ciudad Real provinces, Castilla-La Mancha region, Spain.

Participants who had valid data on ADHD-RS-IV [13], completed by parents and teachers simultaneously, were included in the current study (*n* = 1189).

### Procedures

From the Regional Department of Education and Science of Castilla-La Mancha, Spain, a letter was sent to each of the selected schools to inform of the purpose of our study. Subsequently, the researchers explained the objectives and methods of the study to the management of the school to obtain the consent of the school board. With the help of the teachers, a letter was sent to all the parents inviting them to a meeting at the school, the objectives, measures and procedures of our study were explained, solving the questions and doubts of the parents. Signed informed consent was obtained from all parents for the participation of their children in the study and in addition children gave their verbal consent. Later, a researcher distributed the rating scales (parents’ and teachers’ versions) in the schools. Parents and teachers completed the questionnaire and 1 week later returned them to the research team. A total of 1604 closed packets were distributed, and 1437 parents and 1515 teachers returned them (89.6% and 91.4% respectively). The study protocol was approved by the Clinical Research Ethics Committee of the Virgen de la Luz Hospital in Cuenca and the General University Hospital in Ciudad Real and by the Ministry of Education and Science of the Regional Government of Castilla-La Mancha, Spain (FIS PI12/00761).

### Measures

#### Attention-deficit/hyperactivity disorder symptoms

The parents’ and teachers’ versions of the ADHD-RS-IV [13] were used. This questionnaire has a large base of normative data and demonstrated validity and reliability in children and adolescents [17, 27, 28]. The ADHD-RS-IV Spanish preschool’s version is an 18 item scale, with each item corresponding to one of the 18 DSM-IV diagnostic criteria and can be completed by either parents (home version) or teachers (school version) [17]. The scale is distributed among three dimensions: inattention symptoms (nine items), hyperactivity/impulsivity symptoms (nine items) and total (18 items). The respondent rates each item on a Likert score from 0 (never or rarely) to 3 (very often), where higher scores indicate greater frequency and intensity of ADHD symptoms. The scale provides scores for inattention symptoms, hyperactivity/impulsivity symptoms and total score.

#### Case definition

The 90th percentile cut-off was used to establish the prevalence of inattention, hyperactivity/impulsivity and combined subtype symptoms, by age groups: 4 and 5 [29] and 6 years-old [13] according to the age ranges established by the American Academy of Pediatrics for the diagnosis of ADHD [30]. This cut-off was proposed by DuPaul et al. [13] for the ADHD-RS-IV scale, and it is widely used in other studies which allow comparability [18, 31, 32]. It was considered that a child was with probable ADHD inattention symptoms or hyperactivity/impulsivity symptoms when both parents and teachers scored ≥90th percentile on this scale. It was considered that a child was with probable ADHD symptoms when both parents and teachers scored ≥90th percentile on the total scale (combined subtype). The total prevalence was calculated by adding the values of the three subtypes symptoms (inattention, hyperactivity/impulsivity and combined).

#### Socio-demographic variables

Age, sex, school (public and private), origin (native-children born in Spain- or foreign - children or one of their parents born outside of Spain) and SES were collected from a questionnaire for parents.

#### Family SES

Data regarding family SES were gathered by using self-reported occupation and education questions completed by either parent. Paternal and maternal education were classified separately as was primary education (functionally illiterate, with no education or those who had not completed primary education), middle education (primary education, high school/secondary education or ‘Bachillerato’), and university education (university degree or PhD). Parental occupation was classified into five categories as follows: (i) supervisor/manager or freelance with ten employees or more; (ii) supervisor/manager or freelance with less than ten employees; (iii) freelance with no staff; (iv) non-qualified staff and unskilled worker; and (v) household chores, unemployed and others. An index of SES was calculated using the items regarding parents’ education and occupation [33]. According to the scale proposed by the Spanish Society of Epidemiology, this index distinguishes five categories of family SES: lower, upper-lower, lower-middle, upper-middle and upper. However, since there were very few participants in the categories at the extremes, we have regrouped these into three categories: low (lower and upper-lower), middle (lower-middle) and high (upper-middle and upper).

#### Statistical analyses

The associations of probable ADHD and subtypes symptoms with age-groups (4 and 5 or 6 years-old), sex, school, family SES and origin of participants were assessed using the Chi-squared test. In addition, the agreement between the two informants (parents and teachers) was evaluated for each disorder subtype using the Cohen kappa coefficient. Kappa values 0–.20 were considered slight, .21–.40 fair, .41–.60 moderate, .61–.80 substantial and .81–1 excellent [34]. Statistical analyses were carried out using IBM SPSS Statistics 22.0 and EPIDAT 4.1.

## Results

We invited 1604 children to participate in the study and 1189 had valid data (74.0%), of which 575 (48.3%) were girls. The age of participants ranged from 4 to 6 years-old (mean = 5.30, *SD* = .60) and 18.8% lived in the provincial capitals. No differences in age, sex and family SES were found between children who agreed to participate and those who did not. Distribution of preschoolers according to age, sex, school type, origin and family SES are depicted in Table 1.

**Table 1:** Characteristics of the sample (*n* = 1189)

	Number	Percent
Age, years		
4	93	7.8%
5	646	54.4%
6	450	37.8%
Sex		
Boys	614	51.6%
Girls	575	48.4%
School		
Public	1047	88.1%
Private	142	11.9%
Origin		
Spanish	1022	86.0%
Foreign	167	14.0%
Family socio-economic status		
Low	346	29.2%
Middle	538	45.2%
High	305	25.6%

The agreement between parents and teachers for each ADHD subtype symptoms scale showed the following Cohen’s kappa coefficient estimations: hyperactivity/impulsivity symptoms (κ = .087), inattention symptoms (κ = .221), and total prevalence (κ = .162). Thus, the estimates for hyperactivity/impulsivity symptoms could be considered as poor agreement, and for inattention symptoms as fair agreement.

The associations of probable ADHD and subtypes symptoms with age-groups (4 and 5 or 6 years-old), sex, school, family SES and origin of participants from 90th percentile are shown in Table 2. Overall, the prevalence of probable ADHD symptoms in our population was 5.4% (2.6% inattention subtype symptoms, 1.5% hyperactivity/impulsivity subtype symptoms and 1.3% combined subtype symptoms). Significant differences in prevalence rates between parents and teachers (23.9% vs. 12.9%, *p* < .01) were found, with the highest difference in the hyperactivity/impulsivity subtype symptoms (10.5% vs. 3.9%, p < .01). Regarding age, children aged 4 and 5 years showed a higher prevalence of probable ADHD symptoms in inattention subtype symptoms and total (sum of all subtypes symptoms) than children aged 6 years. Also, children with low family SES reported higher prevalence of probable ADHD symptoms than medium and high family SES in all subtypes symptoms and total (sum of all subtypes symptoms).

**Table 2:** Prevalence of probable ADHD and subtypes symptoms by sex, school, origin and socio-economic status (90th percentile)

		Combined		Inattention		Hyperactivity / Impulsivity		Total^a^	
	Total N	% (N)	*P* Value	% (N)	*P* Value	% (N)	*P* Value	% (N)	*P* Value
Parents Prevalence		4.3 (51)	**< 0.01**	9.1 (108)	**< 0.01**	10.5 (125)	**< 0.01**	23.9 (284)	**< 0.01**
Teachers Prevalence		2.0 (24)		7.0 (83)		3.9 (47)		12.9 (154)	
Prevalence^b^									
Age 4 to 6	1189	1.3 (15)		2.6 (31)		1.5 (18)		5.4 (64)	
Age, years									
4 to 5	739	1.6 (12)	0.15	3.5 (26)	**0.01**	2.0 (15)	0.06	7.2 (53)	**< 0.01**
6	450	0.7 (3)		1.1 (5)		0.7 (3)		2.5 (11)	
Sex									
Boys	614	1.3 (8)	0.89	2.9 (18)	0.47	1.3 (8)	0.54	5.5 (34)	0.89
Girls	575	1.2 (7)		2.3 (13)		1.7 (10)		5.2 (30)	
School									
Public	1047	1.4 (15)	0.15	2.9 (30)	0.13	1.7 (18)	0.12	6.0 (63)	0.09
Private	142	0.0 (0)		0.7 (1)		0.0 (0)		0.7 (1)	
Origin									
Spanish	1022	1.4 (14)	0.41	2.5 (26)	0.73	1.7 (17)	0.30	5.6 (57)	0.98
Foreign	167	0.6 (1)		3.0 (5)		0.6 (1)		4.2 (7)	
Socio-economic status									
Low	346	2.6 (9)		4.9 (17)		2.9 (10)		10.4 (36)	
Middle	538	0.6 (3)	**0.03**	1.7 (9)	**< 0.01**	0.7 (4)	**0.04**	3.0 (16)	**< 0.01**
High	305	1.0 (3)		1.6 (5)		1.3 (4)		3.9 (12)	

^a^Sum of all subtypes, ^b^Parents and teachers scored ≥ 90^th^ percentile; in bold when *p* < 0.05

## Discussion

Our findings suggest that the prevalence of probable ADHD symptoms in children among aged 4 to 6 years was 5.4% (2.6% inattention subtype symptoms, 1.5% hyperactivity/impulsivity subtype symptoms and 1.3% combined subtype symptoms). Also, younger children and those with low family SES reported a higher prevalence of probable ADHD symptoms than older children and those with medium and high family SES.

According to parents, 23.9% of children were with probable ADHD symptoms. By contrast, teachers reported a value of 12.9%. The significant difference in the values of prevalence given by parents and teachers may be due to environmental expectations, behavioural differences in children in different contexts and the possibility of comparisons with classmates. The poorer health status perceived by mothers of ADHD-diagnosed children, as of social dysfunction and anxiety, can be related to the clinical manifestations of hyperactivity which are more frequently reported by the family [20]. In line with Amador-Campos et al. [35] and Canals et al. [20], we have an overestimation of the hyperactivity/impulsivity subtype symptoms in parents [20, 35]. Looking at the data and the ADHD-RS-IV scale, in the teachers’ version few 6-year-old girls reported prevalence of ADHD symptoms; this is due to the large difference in the cut-off points between both scales and age groups. Also, the teachers’ cut-off points in relation to hyperactivity and inattention symptoms are about 25% higher than those in parents, except in girls aged 4 and 5 years; this involves obtaining higher values of prevalence from parents. For this reason, and in accordance with recommendations, ADHD diagnosis was considered when at least two informants reported symptoms of any ADHD subtype: parents and/or teachers and/or clinician.

The reviews that have examined the prevalence of children with probable ADHD symptoms have reported large differences in their estimates among countries [2, 14, 16, 36]. This variability could be explained by the instruments used to measure ADHD symptoms (questionnaires or interviews), and the diversity of age-range or environmental characteristics. In the Spanish context, our estimates of prevalence of ADHD in children aged 3–6 years (5.4%) are similar to those reported by a previous study [20]. Studies in 4 and 5 years-old children from Colombia (6.2% to 18.2%) [21, 37] and Japan (7.0%) [22] showed also similar or higher prevalence of probable ADHD symptoms than ours (7.2%). Moreover, in 6 years-old children, Spanish and international studies reported higher rates of probable ADHD symptoms than ours (2.5%).

In line with our results, several studies have reported a higher prevalence of inattention subtype symptoms than the other ADHD subtypes [21, 38]. However, other studies reported a higher prevalence of combined subtype symptoms [18, 39]. Studies reveal that the frequency and intensity of symptoms of inattention are common in primary education [21, 40]. The inattentive children are recognised when teachers perceive that they are having a lot of difficulty staying focused on tasks, remembering what they have read or in keeping up with their work in school [23].

In comparison to earlier studies [16, 18, 41], our results showed a higher prevalence of probable ADHD symptoms in preschoolers in the inattention subtype symptoms and total (sum of all subtypes symptoms) than in older children with higher percentages in the parents’ questionnaire. In addition to the natural history of the disorder [42], it is possible that the transition from kindergarten to primary education, by an additional increase in maturation, can make children more aware of the rules for classroom behaviour, thereby facilitating greater adherence to them in older children compared with younger. The difficulty of diagnosis in preschoolers should also be taken into account, so it is likely that common behaviours of children aged 4–5 years (such as difficulty sitting still, paying attention or controlling impulsive behaviour), might be confused with ADHD symptoms.

The extent to which the prevalence of ADHD symptoms and its subtypes varies by family SES is also unclear. Although an elevated ADHD symptoms prevalence is described in lower SES populations [21, 41, 43], other studies have not observed a difference among SES categories [19, 44, 45]. Our findings showed differences between family SES categories (10.4% low level, 3.0% middle level and 3.9% high level). Possible reasons for those differences include family dysfunction, child abuse and poor educational conditions associated with low SES [46]. Moreover, Froehlich et al. [41] have argued that etiological factors of ADHD such as tobacco exposure and complications of pregnancy and delivery, might partially explain these differences among socio-economic groups [41].

Although the prevalence of probable ADHD symptoms in boys is usually higher than in girls [3, 41], our results, and those of other authors [18, 22], do not confirm these differences by sex.

As far as we know, several studies that compared both types of schools (public and private) [24, 25, 47], have reported a higher prevalence of children with probable ADHD symptoms in public schools than in private ones. Our findings did not show differences between these types of schools, however, there is a trend towards a greater prevalence of ADHD symptoms in public schools. This may be justified by the fact that the results showed few children in private schools with low SES (12 children; 7.2% of total), considering that children with low SES have a higher prevalence of ADHD symptoms, there seems to be a reasonably low rate of ADHD symptoms in this type of school.

This is the only study that analysed differences between nationalities (native versus foreign samples) and no difference was reported [23]. Confirming this result, our findings showed no differences between Spanish and foreign children. Biederman and Faraone [7] explained that the low prevalence of ADHD symptoms in immigrant children might be due to cultural differences, which benefit from higher tolerance for ADHD symptoms making it even more difficult to diagnose [7].

### Strengths and limitations

There are a number of strengths to this study as compared to others published: (i) the prevalence of children with probable ADHD symptoms was calculated through the two versions (parents and teachers) of a validated scale, according to children’s age and percentile; (ii) we obtained a high rate of response from parents and teachers; and (iii) this is the first study that measured the prevalence of probable ADHD symptoms in 4–5 years-old Spanish children in the region of Castilla-La Mancha, Spain.

Potential limitations should be taken into consideration: (i) we did not record whether children were taking medication for ADHD; (ii) we do not know whether there are any pre-existing diseases such as learning disabilities or global delays, or if participants do aerobic exercise that could affect the prevalence of being probable ADHD symptoms [48]; (iii) we did not verify suspected ADHD symptoms through interviews and/or testing conducted by health professionals (psychiatrist or psychologist); and (iv) given that the data comes from a study that had other aims, and representativeness of the sample could not have been achieved. It should be noted that the region where the study took place is uniform in terms of demographic characteristics, and almost all the children, especially in the towns, are in public education, so the representativeness of the sample might be guaranteed.

## Conclusions

The prevalence of probable ADHD symptoms in Spanish children aged 4–6 years is 5.4%. Children aged 4–5 years and those who belong to low SES have a higher prevalence of probable ADHD symptoms than children aged 6 years and a medium-high family SES.

Our findings suggest that a significant percentage of preschoolers are with probable ADHD symptoms, thus an early identification and an understanding of the predictors of being probable ADHD symptoms are needed to direct appropriate identification and intervention efforts. These efforts should be especially addressed to vulnerable groups, particularly low SES families and younger children.

## Abbreviations

ADHD RS-IV: Attention Deficit Hyperactivity Disorder Rating Scales IV
ADHD: Attention-Deficit/Hyperactivity Disorder
SES: Socio-economic status
